# Simulation Training to Improve Informed Consent and Pharmacokinetic/Pharmacodynamic Sampling in Pediatric Trials

**DOI:** 10.3389/fphar.2020.603042

**Published:** 2020-12-11

**Authors:** Bjoern B. Burckhardt, Agnes Maria Ciplea, Anna Laven, László Ablonczy, Ingrid Klingmann, Stephanie Läer, Karl Kleine, Michiel Dalinghaus, Milan Đukić, Johannes M. P. J. Breur, Marijke van der Meulen, Vanessa Swoboda, Holger Schwender, Florian B. Lagler

**Affiliations:** ^1^ Institute of Clinical Pharmacy and Pharmacotherapy, Heinrich Heine University, Düsseldorf, Germany; ^2^ Pharmabrain Research and Training Center, Berlin, Germany; ^3^ Göttsegen György Hungarian Institute of Cardiology, Budapest, Hungary; ^4^ Pharmaplex Ba, Wezembeek‐Opperm, Belgium; ^5^ Simply Quality–Dr. Karl Kleine, Weilheim in Oberbayern, Germany; ^6^ Erasmus MC, Sophia Children’s Hospital, Rotterdam, Netherlands; ^7^ University of Belgrade, Medical School, Belgrade, Serbia; ^8^ University Medical Center, Wilhelmina Children’s Hospital, Utrecht, Netherlands; ^9^ Department of Paediatrics and Adolescent Medicine, Medical University of Vienna, Vienna, Austria; ^10^ Mathematical Institute, Heinrich Heine University, Düsseldorf, Germany; ^11^ Department of Pediatrics, Institute for Inherited Metabolic Diseases, Paracelsus Medical University, Salzburg, Austria

**Keywords:** pediatrics, communication, study conduct, pharmacokinetic/pharmacodynamic, patient recruitment, simulation training, clinical study

## Abstract

**Background**: Pediatric trials to add missing data for evidence-based pharmacotherapy are still scarce. A tailored training concept appears to be a promising tool to cope with critical and complex situations before enrolling the very first patient and subsequently to ensure high-quality study conduct. The aim was to facilitate study success by optimizing the preparedness of the study staff shift.

**Method:** An interdisciplinary faculty developed a simulation training focusing on the communication within the informed consent procedure and the conduct of the complex pharmacokinetic/pharmacodynamic (PK/PD) sampling within a simulation facility. Scenarios were video-debriefed by an audio-video system and manikins with artificial blood simulating patients were used. The training was evaluated by participants' self-assessment before and during trial recruitment.

**Results:** The simulation training identified different optimization potentials for improved informed consent process and study conduct. It facilitated the reduction of avoidable errors, especially in the early phase of a clinical study. The knowledge gained through the intervention was used to train the study teams, improve the team composition and optimize the on-ward setting for the FP-7 funded “LENA” project (grant agreement no. 602295). Self-perceived ability to communicate core elements of the trial as well as its correct performance of sample preparation increased significantly (mean, 95% CI, *p* ≤ 0.0001) from 3 (2.5–3.5) to four points (4.0–4.5), and from 2 (1.5–2.5) to five points (4.0–5.0).

**Conclusion:** An innovative training concept to optimize the informed consent process and study conduct was successfully developed and enabled high-quality conduct of the pediatric trials as of the very first patient visit.

## Introduction

Despite the European Pediatric regulation aiming to bring evidence-based drug therapy to the vulnerable pediatric population, its effects are still lacking (Samiee-Zafarghandy et al., 2014). Even 13 years after the regulation got into force, there is still a high demand for well-investigated drug therapies and tailored medication for the pediatric population. Highly required pediatric trials to add missing data for evidence-based pharmacotherapy are still scarce for this vulnerable population.

About 25% of randomized controlled trials in adults and up to 40% of pediatric trials are discontinued prematurely (Kasenda et al., 2014; Schandelmaier et al., 2017) because of insufficient patient recruitment (Pica and Bourgeois, 2016). Parents are more reluctant to consent on behalf of their children than for themselves (Caldwell et al., 2003), making recruiting for pediatric trials challenging. Key elements are the quality of the informed consent process (Koyfman et al., 2016), and a well-planned and well-organized study conduct (Pica and Bourgeois, 2016). In particular, avoidable errors occur mainly at the beginning of a clinical study. As a consequence, potentially eligible patients get lost in the early phase of the study (Taekman et al., 2010).

Simulation training in healthcare (Chopra et al., 1994; Schwid et al., 2002; Windsor et al., 2008) has shown that critical steps in complex situations can be improved (Shapiro, 2004; Wright et al., 2005). Simulating experiences with patients and relatives, and systematically analyzing these experiences prevents avoidable mistakes in practice. The principle is well established in medical education in many areas. Simulation training improves teamwork and critical thinking within clinical teams. The training has demonstrated its usefulness by increased guideline compliance and fewer process deviations (Shapiro, 2004; Windsor et al., 2008; McGaghie et al., 2011). Simulation training for the preparation of a clinical trial is a promising tool that appears to be robust to improve study protocol compliance, communication and study conduct. It is an auspicious tool to ensure high-quality study conduct to cope with critical and complex situations before enrolling the very first patient.

In the context of the pediatric studies within the LENA project (Labeling of Enalapril from Neonates up to Adolescents, FP7/2007-2013 Grant Agreement No. 602295) (Bajcetic et al., 2019) protocol compliance, obtaining informed communication and optimal study conduct were considered challenging. According to the agreed Pediatric Investigation plan, seventy percent of patients were mandatory to be younger than one year of age. Therefore, challenging recruitment and tough blood sampling were likely to occur within the LENA project, and avoidable errors were supposed to cause a substantial negative impact.

This study-specific prerequisite of the LENA project motivated us to develop and conduct a tailored simulation training in preparation for the clinical trials. This manuscript describes how this simulation-based training for study teams was developed and applied, which experiences were gained and how the participating study teams assessed the effects. Thus, it allows other groups to apply our comprehensive, patient/parent centered and innovative approach to their own clinical trials.

## Methods

### Pedagogical Principles

The aim of the intervention was to facilitate study success by optimizing the preparedness of the study staff, in other words to shift the learning curve with critical study elements toward the pre-study period. Thus, the training intervention should enable -on the one hand- practical experience and familiarization of the study staff and on the other hand, allow for optimization of the study procedures and documents toward the study staff’s needs in practice. The structure and setting of medical simulation team training seemed promising for these aims. The conceptual framework, which best describes this type of experiential learning is Kolb’s learning circle (Kolb, 2014). It is characterized by the four learning phases: concrete experience, reflection, conceptualization, and experimentation. In our training this was implemented by simulation scenarios, in which study staff gathered experience with communication challenges or pharmacokinetic/pharmacodynamic (PK/PD) sampling procedures (= concrete experience). This experience was recapitulated in the structured scenario debriefing which included the reviewing of selected video sequences of the scenario (= reflection). Moreover, potential improvements were identified. Plans on how to implement these improvements (e.g., by reorganizing the teamwork, adjusting the procedures etc.) were built (= conceptualization). These plans were then applied and tested in the consecutive scenarios (= experimentation). The process of briefing, scenario, debriefing with development of an improvement plan and application of the improvements within the consecutive scenario was repeated iteratively. The practical application of this didactic principle within our training is further illustrated in Supplementary Material S1. All participants followed all scenarios and participated in all debriefings, thus learning was not limited to the own hands on experience but included also learning based on the observation and debriefing of other scenarios. The debriefing was facilitated by simulation and communication trainers using methods and a structure recommended for trainings of technical skills, teamwork training, and inter-professional collaboration (Eppich and Cheng, 2015). This PEARLS method helps to facilitate an active, collaborative, self-directed and learner-centered learning. Thus it was very useful for leveraging our participants’ high level of clinical trial experience for improving individual and team competencies as well as developing study site specific plans for mastering the critical study elements. Amongst other benefits, this innovative training approach stands out over unidirectional information transfer or simple repetitive drills as it enables study teams to find and internalize how the study protocol can be best realized in the working environment and situation of their site. This is particularly helpful with regard to the diversity of study sites commonly found in international multi-center trials.

### Learning Environment

The simulation intervention was part of the overall training concept for the LENA pediatric trials that is depicted in the Supplementary Material S2 and described in detail elsewhere (Ciplea et al., 2018). For the 1.5-days training, the investigators, research coordinators, and study nurses of all sites visited the Medical Simulation Center Salzburg (Austria), three months before the expected “first patient first visit”. The setup comprised a training room reflecting the typical working environment at the corresponding study sites. For this purpose, the equipment, setting, room size etc. at the participants’ sites was investigated in advance and reconstructed by props, paravents, clinical and lab equipment. Further, a one-way mirror control booth was used for observation and assessment by the simulation operators and in a debriefing room all delegates currently not actively involved observed the scenario via live streaming. Parents and siblings were role-played. The simulation manikins were prepared to allow for the sampling of artificial blood. For sample processing procedures, blood substitutes and authentic tubes, stickers, and forms of the LENA clinical trials were utilized to achieve high-fidelity.

### Training Objectives and Scenario Development

Great importance was attached to the identification of the most relevant training objectives and the design of highly realistic scenarios, not only to address the most critical study elements but also to achieve a high level of training motivation and success. An inter-professional, interdisciplinary faculty of eleven members with expertize in the field of pediatrics, Good Clinical Practice (GCP), trial management, communication training, bioanalysis, clinical pharmacy, and patient representatives was formed. For the identification of critical challenges, a pre-mortem analysis was used (Mitchell et al., 1989), starting with the hypothetical assumption that a project has failed, and working backwards to determine what could have potentially lead to the failure of the project. This process identified the following potential threats as most relevant to the successful conduct of the pediatric LENA trials: 1) a very young (70% < 1 year of age) and very vulnerable target population with severe cardiac disease 2) frequent visits and 12 months duration of study participation, 3) a high number of blood samplings with 4) complicated pre-analytical procedures. “Communicating the importance of the trials and key trial elements with parents, clinical and study staff” and “blood sampling and processing” were consequently defined as most critical study elements. Obviously, these are most important in any PK/PD study, however the identification of the potential threats allowed the deduction of very relevant and realistic scenarios as well as concrete training objectives. Developed scenarios and objectives are summarized in Table 1.

**TABLE 1:** Learning objectives.

	Learning objectives
Scenario 1: Parents do not see the need for the study or find the requirements too rigorous	Establish a setting for the communication that is quiet, friendly and not associated with previous “bad news” experiences of the parents
	Identify and eliminate risks like emotional states that prevent information transfer and subsequently successful communication
	Communicate the expected benefits of the mini tablets (IMP) over the currently used formulation of enalapril (e.g., facilitated by demonstrating the mini tablets) and the expected burden and risks involved in study participation in a language that meets the parents’ needs
	Detect motives for a rejection of research in general or the proposed study in particular and provide relevant information in a patient-centred and empathic way
Scenario 2: Parents are not able to follow the informed consent discussion	Learning objectives 1‐3 plus
	Identify barriers to comprehension and use supportive material like picture card etc.
	Facilitate parental questions and expression of concerns
	Prioritize on key information with highest importance for parents
	Schedule a follow-up appointment, if needed
Scenario 3. Lack of a trustful relationship with the parents	Learning objectives 1‐3, 6 plus
	Identify barriers to trust
	Use confidence-building body language and positive vocabulary (e.g., “research” instead of “clinical trial”)
	Actively involve study nurse, referring physician with lower barriers or pre-existing strong relationship to parents
Scenario 4: Fear of invasive medical procedures	Learning objectives 1‐3, 6 plus
	Identify reasons for fear without downplaying or concealing risks and burdens
	Explain how risks and burdens are kept to a minimum
	Point out that the study protocol has been approved by the ethics committee.
Scenario 5: Uncooperative clinical staff	Learning objectives 1‐3
	Identify causes of negative reactions and address each single concern in a respectful way
	Define benefits and justify risks and burdens for participants and efforts for clinical and study staff
	Explain how risks, burdens and efforts are kept to a minimum
	Apply conflict management methods
Scenario 6: Lack of confidence in the process by members of the study staff	Learning objectives 1‐3
	Identify the exact cause behind the lack of confidence
	Evaluate the need for information and provide it in respectful way
	Make own personal beliefs about the study transparent
	Use self-confident and positive wording and body language

### Training Format

The 1.5-days training was structured in three parts. In part one, presentations were given on key elements of the study (e.g., number of visits, frequency of blood drawings etc.) by a pharmacist, on communication techniques by a communication trainer with specific health care background, on PK/PD sampling and documentation procedures by a pharmacist as well as on the simulation approach by the training director. Furthermore, the participants familiarized themselves with the “LENA”-study specific documents and equipment (e.g., orodispersible mini-tablets, the informed consent form, sampling and documentation material) and the training environment. Part two and three took place at the consecutive day. In part two, PK/PD procedures were simulated. The study teams had to perform the blood sampling, the complex and time-critical pre-analytic procedures to be performed on ward as well as the comprehensive documentation under realistic conditions and in strict accordance to the study protocol. As many different tubes per patient and time point were needed and a deviation from protocol could render the samples invalid, workload as well as time and success pressure of this task were high. The participants were guided stepwise from practical demonstrations over deliberate practice of single tasks toward full runs of sampling and pre-analytic procedures in the simulation scenarios. Within two to three rounds, the difficulty of scenarios was gradually increased to a realistic setting, which also included the simulation of stressors like emotionalized parents or interrupting clinical staff by actors. Sampling, labeling, centrifuging, pipetting, cooling, documentation and keeping the time limits were meticulously monitored in every scenario. Within debriefing, observed deviations from protocol, potential reasons for failure and potential for improvement were worked out in a learner-centered manner.

In the third and last part, scenarios with communication challenges were conducted. The participants could select the situation of their scenarios between informed consent conversation or critical communications with either clinical staff or study staff. However, the particular tasks and challenges of the scenarios (see Table 1) were not disclosed to the participants in advance. Parents and siblings were role-played and to add complexity distractors such as crying manikins, urgent telephone calls, annoying behavior of siblings were added. The conversations were conducted according to the standard situation of the participants, which included teams of investigator plus research nurse and of principal and sub-investigators. Participants could speak in their mother language or English. Where needed, the communication trainer was supported by a simultaneous translator. Different to the PK/PD scenarios, the performance could not be dichotomously classified correct or incorrect. Thus, the debriefing focused on how successful communication strategies were used in the respective scenario and what could be improved. It was encouraged to use lay language, open-ended questions, active and reflective listening including positive body language, without interrupting, showing empathy and compassion, and joint decision-making to ensure that the patient’s preferences, needs and values were respected.

### Prospective Evaluation of the Simulation Training by Self-Assessment Survey

As all study teams should benefit by this training intervention, a controlled study to proof its effects was not feasible within this project. Thus, we conducted a prospective survey study to assess the pre-training ability and preparedness of participants as well as the perceived training impact on knowledge, attitudes and working practice. The survey study was approved by the ethical committees of Salzburg state government, Austria (study no. 415-E/1909/2–2015) and Heinrich Heine University Duesseldorf, Germany (study no. 5138). Training participants were surveyed at four time points: Before and directly after the training to assess pre-training competencies and immediate training effects, and at study start plus upon 70% of planned patients’ recruitment to determine if the training effects were still present when needed and how they were rated after applying the trained skills. Details on the content of each questionnaire are provided in Supplementary Material S3. The five-point Likert-Scale (1 = very poor/strongly disagree, 5 = very good/strongly agree) ratings were evaluated by a Wilcoxon test using the statistical software environment R (version 3.3.3). The Wilcoxon signed-rank test was applied to the differences of the pre- and post-training competencies. For each test, a statistical level of significance of *p* = 0.05 was considered.

### Post-Hoc Analysis of Participant Recruitment and Protocol Compliance in Pharmacokinetic/Pharmacodynamic Sampling

Encouraged by the positive feedback on the training intervention and to understand if we succeeded in shifting the learning toward the pre-initiation period, we performed a post-hoc analysis of learning effects within our trials. For this purpose, the rate of recruitment for all sites over the complete recruitment period was analyzed. Additionally, the number of PK/PD samples with protocol deviations during the first three months was analyzed in comparison to the whole 18-months observation period via an eCRF evaluation.

## Results

### Experience Gathered

In four 1.5-days trainings between July and October 2015, twenty-three participants from six study sites were trained. In total, 13 (56.5%) physicians and 10 (43.5%) (study) nurses joined the training. 60.9% of the participants had more than 5 years of experience in clinical studies. Details on the demographics of the participants are provided in Supplementary Material S4. The scenarios were conducted in Dutch (Samiee-Zafarghandy et al., 2014), English (Koyfman et al., 2016), German (Samiee-Zafarghandy et al., 2014), Hungarian (Kasenda et al., 2014), and Serbo-Croatian (Schandelmaier et al., 2017). When requested to select between communication challenges with the informed consent situation or critical situations with concerned clinical or study staff, all teams decided for the first.

### Needs and Plans for Improvement Identified With the Scenarios

Although in general, the competency of the participants in providing information on the key aspects of the trials in a patient/parent centered manner was good, some reoccurring opportunities for improvement may also be relevant for other projects.

Lay language was used in all conversations, but study elements were mostly described in very technical medical language. It was encouraged to aim for a balanced speaking time (50:50) between parents and study team, to answer all questions of the parents and to keep the focus on practical study elements (e.g., participants’ obligations, benefits, risks etc.) rather than scientific background information. Only two participants achieved balanced speaking time. In average, health care professionals (HCPs) spoke 66 ± 14% of the time. Interestingly, in conversations strongly dominated by HCPs, the focus tented to lay on scientific terms and parental questions remained unanswered partly. Intendedly added stressors like annoying siblings or uncooperative parents strongly impacted the conversation. Even if HCPs obviously were aware of the situation and tried to counteract specifically, most were surprised to see within the debriefing how these stressors that they face commonly in pediatrics, impacted them up to aggressive emotions. The practical demonstration of the easy-to-use orodispersible mini-tablets was perceived very helpful to illustrate the study benefits. Upon reflection and discussion of these experiences most participants planned to put more emphasis on creating a positive situation and prevent stressors, e.g., by organizing an extra person to look after siblings or by scheduling an additional appointment. Moreover, several participants concluded to change their approach for these conversations from a mainly unidirectional description of all study details toward a very brief introduction of the key study elements immediately followed by more active involvement of the parents and addressing their questions.

In the first round of PK/PD sampling, 62.5% of all samples were prepared in accordance with the protocol. Exceeding the time limits, incorrect sample preparation and incomplete documentation were the main issues. Potential sources for protocol deviations revealed by video-assisted reflection of the scenarios included under-staffing, sub-optimal arrangement of the setting, insufficient assignment of tasks and communication affecting teamwork in general but particularly the hand-over between sampling and processing. Thus, for the consecutive rounds team size was increased from two to three or four members, responsibilities were shared, and the working environment was refined. Although in round two distractors like crying children, parents asking questions and emergency calls were integrated to increase realism and stress, the quality of tasks improved substantially. The strict timelines between sampling to sample freezing for PD parameters of ≤15 min were met in more than 91%. The final comprehensive sample preparation increased to 97.4% in average, while trial documentation was accurate in 100%.

### Results of the Self-Assessment Survey

**FIGURE 1:**
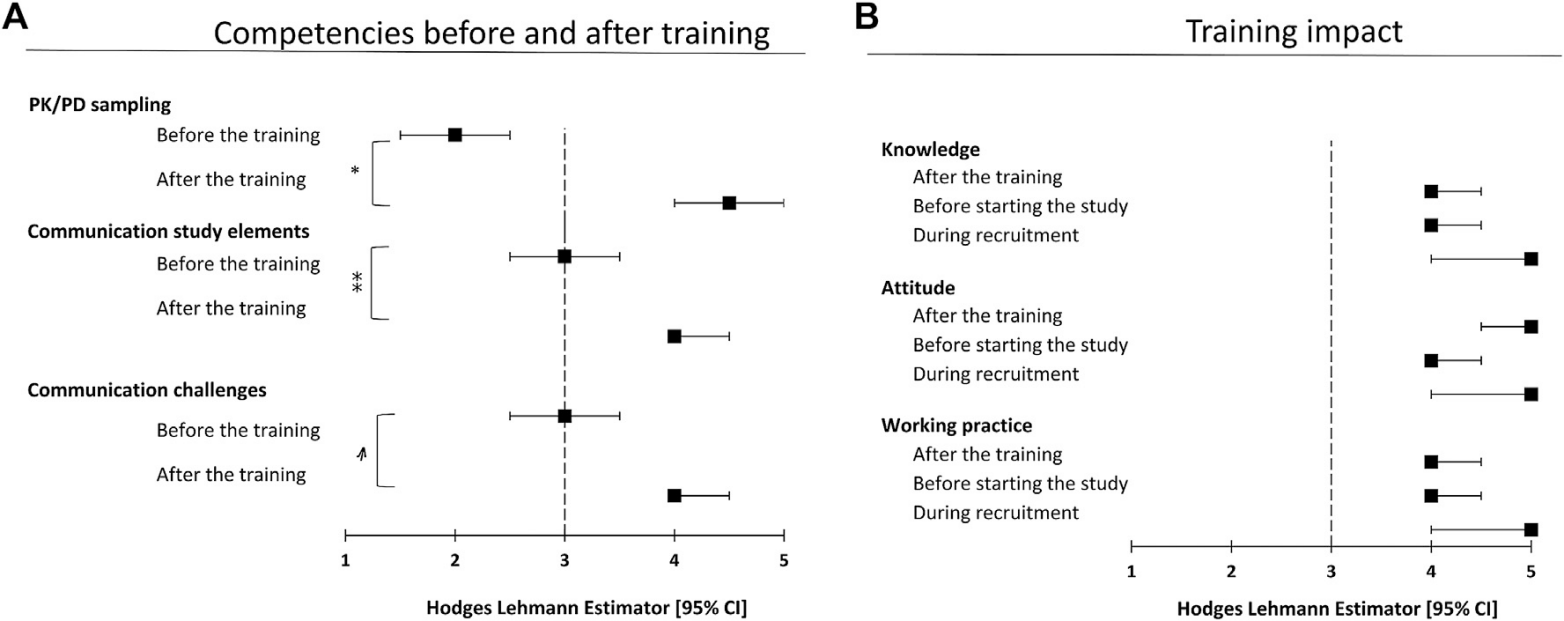
Results of the conducted self-assessment survey. Panel **(A)** depicts the participants rating on their competencies before and two days after the training concerning communication and PK/PD sampling. Results are depicted as Hodges Lehmann Estimator (HLE) with corresponding CI using a 5-point Likert-scale (1 = very poor, 5 = very good, *p < 0.0001, **p = 0.0001, *p* = 0.0002). Panel **(B)** illustrates the perceived long-term effects on attitude, knowledge, and working practice. Time points of determination were immediately after the simulation training (“after the training”), short before individual study start at each site (“before starting the study”) and at time point of 70% of anticipated study-wide patient recruitment (“during recruitment”).

All 23 participants of the training consented to take part in the survey-based evaluation. The response rate was 100% at the first three time points and 95% at the last. The total follow-up time ranged between 19 and 23 months after the simulation training. Both, communication skills as well as trial-specific sampling and sample preparation abilities were significantly higher self-assessed immediately after training (Figure 1A).

The self-assessment survey indicated a significant increase of the ability to communicate the core elements of the clinical trial (*p* = 0.0001) and the preparation to deal with trial-related communicative challenging situations (*p* = 0.0002). The abilities regarding sampling and sample preparation procedures improved (*p* < 0.0001) toward “good” (scale level 4) or “very good” (scale level 5) after the training (Figure 1A). The training's impact on knowledge, attitude and working practice was rated “good” or “very good” at all time points of assessment (Figure 1B). The approach of simulation-based training in preparation for clinical trials was unanimously perceived as suitable.

### Post-Hoc Analysis of Participant Recruitment and Protocol Compliance in Pharmacokinetic/Pharmacodynamic Sampling

**FIGURE 2:**
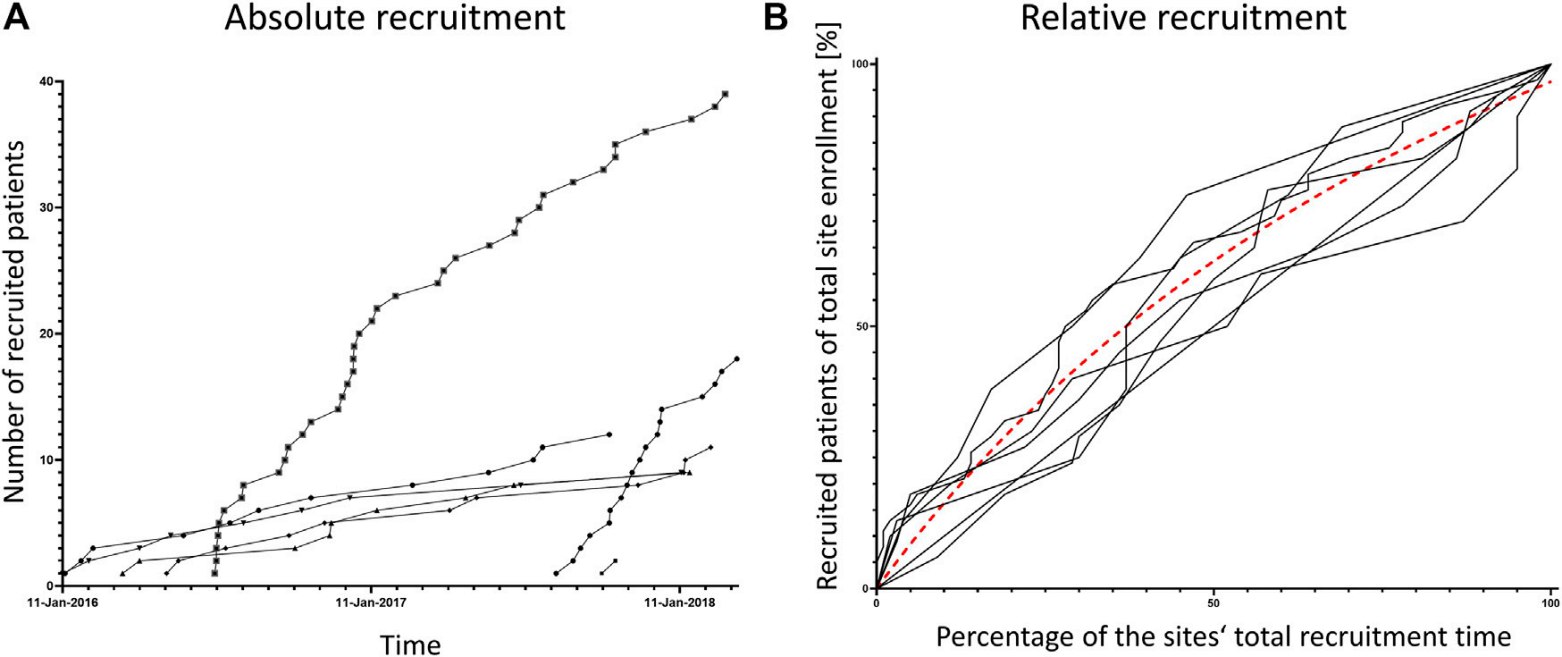
Recruitment rates per site. Both panels show the recruited patients per study site over the total study period. In panel **(A)** each patient is represented by one symbol, patients of one site are connected by lines. Panel **(B)** shows normalized data, the percentage of patients recruited over the percentage of each site’s recruitment time. The dashed line is a non-linear curve fit of all curves indicating that the mean recruitment rate is highest at recruitment start.

Total number of recruited patients and rate of recruitment differed substantially between all sites (Figure 2A). However, all sites showed their top recruitment rate right at start of recruitment (Figures 2 A,B). The evaluation of proportion of PK/PD samples with protocol deviations revealed a very high-quality level in general. All sites started with levels of 95 to 100% of correct samples, which was above or in the range of their overall performance (Table 2) indicating no lag time to top quality rates in PK/PD sampling.

**TABLE 2:** Comparison of PK/PD protocol deviations between early recruitment phase and whole observation period.

			No of PK/PD samples collected	Observed deviations	No of samples obtained according to manual		
Site 1	Early phase of recruitment	1st month	39	2	37	95.0%	88.3%
		2nd month	20	6	14	70.0%	
		3rd month	12	0	12	100.0%	
	Full observation period		228	28	200		87.7%
Site 2	Early phase of recruitment	1st month	26	0	26	100.0%	100.0%
		2nd month	35	0	35	100.0%	
		3rd month	11	0	11	100.0%	
	Full observation period		304	1	303		99.7%
Site 3	Early phase of recruitment	1st month	25	0	25	100.0%	100.0%
		2nd month	27	0	27	100.0%	
		3rd month	12	0	12	100.0%	
	Full observation period		272	0	272		100.0%
Site 4	Early phase of recruitment	1st month	35	0	35	100.0%	100.0%
		2nd month	23	0	23	100.0%	
		3rd month	19	0	19	100.0%	
	Full observation period		172	1	171		99.4%
Site 5	Early phase of recruitment	1st month	140	6	134	95.8%	98.3%
		2nd month	102	1	101	99.0%	
		3rd month	82	0	82	100.0%	
	Full observation period		1,001	7	994		99.3%

Full observation period reflected 18 months (January 2016 until June 2017).

PK/PD, pharmacokinetic/pharmacodynamic.

## Discussion

This is the first report on the development and application of simulation-based training for improving the informed consent process and the PK/PD sampling in clinical trials. Our innovative approach combined a patient/parent centered, interdisciplinary process to identify most relevant protocol specific hurdles and an experiental training program utilizing technical and didactic methods of medical simulation. It allowed us to identify relevant hurdles for successful study conduct, optimize the composition of teams and improve their preparedness, evaluate and improve the feasibility of the study processes and adopt the working environment at the study sites for the expected challenges based on the training experiences. The participants rated the training as very helpful, and their preparedness for the challenges of the study classified significantly higher after the intervention. Moreover, very high quality of sampling and on-ward sample preparation was achieved. This training approach seems a suitable risk mitigation tool and valuable addition to standard methods in clinical trial preparation.

We expected that getting informed consent and PK/PD sampling, including sample processing and its documentation to be the most significant challenges to successful study conduct. On the one hand, this was due to very young and partly critically ill patient population and the complex sampling of the LENA pediatric trials, and on the other hand as a common phenomenon, particularly in trials with minors.

Participant recruitment in timely and targeted manner is a major if not the most important success factor for pediatric and other clinical trials (Denhoff et al., 2015) and the oral communication in recruitment and the informed consent process is of utmost importance (Menon et al., 2012; Treasure and Morton, 2012). It is well shown that there is a relevant probability, that written study information is insufficiently comprehended by most patients (Lagler et al., 2015) and that patients understand study contents better when the information giving physicians receive communication training (Kinnersley et al., 2013; Glaser et al., 2020). It is plausible that the same applies to parents that consent by proxy. Further, according to a large meta-analysis of 72 manuscripts comparing strategies to improve participant recruitment, oral communication on the study intervention was one of two factors with a significant impact on recruitment (Treweek et al., 2018). Nevertheless, doctors often do not receive formal training on how to obtain informed consent (Mason and Allmark, 2000).

Moreover, sampling and processing quality is a major issue for most PK studies. Presumably, over 10 million Euros of funding are lost every year in clinical trials in the European Union due to pre-analytical and analytical problems (Lippi et al., 2016). The vast majority of specimens (over 90%) rejected by laboratories are unsuitable due to incorrect procedures for collection and transportation (Lippi et al., 2016).

The standard method to prepare study staff for a specific study includes monitoring visits and investigator meetings, both showing no or little effect in similar studies (Liénard et al., 2006; Mitchell et al., 2020). It is well known that both, slow participant recruitment and failure to adhere to the protocol are generally most severe at the beginning of trials (Taekman et al., 2010). Besides low familiarity with the protocol and need for “rehearsal”, false self-assessment of the study staff’s preparedness for the challenges of the trial and other human factors as well as weaknesses of the study protocol and data assessment documents and processes seem to impact the learning curve with a clinical trial (Taekman et al., 2010). High-fidelity human patient simulators have previously been used to evaluate the feasibility of study protocols and to train study coordinators in other tasks with significant effects on the confidence of the study staff and indicating overconfidence as a potential risk factor for the study success. The majority of protocol deviation takes place at the beginning of trials (Taekman et al., 2010). In our simulation-based training intervention, we combined protocol and document improvement with the experiential training of our study staff before the trial start. Our approach allowed practical experience and (self-) reflection of the preparedness for the study procedures before the study initiation. Moreover, we gathered valuable information on potential challenges and solutions in the informed consent conversation as well as PK/PD sampling, processing and documentation. The insights and plans for improvement of documents, procedures, teamwork etc. gathered within the training were integrated into the subsequent training in an iterative manner.

According to our self-assessment survey, the training participants felt well prepared for challenges in communication and PK/PD sampling and sample preparation by the training intervention. This favorable judgment of the training effects remained unchanged, even after the majority of patients had been recruited. We are aware of the limitations of self-assessment of competence, and clearly, a controlled study design is needed to confirm the effects of the intervention on the study staff’s competences. Yet, several observations we made throughout the LENA trials are in line with the observed training effects. Although the overall recruitment rate differed between our sites, none showed a low recruitment rate at the beginning of the trial. The rate was quite linear with a tendency to a plateau at the end of the recruitment period. The site with the highest recruitment even showed a logarithmic shape of recruitment over time (Figure 2). Thus, the learning curve effect on recruitment described in many other studies and systematically analyzed by Taekman was not observed (Taekman et al., 2010). Overall, the deviation rate concerning PK/PD sampling and preparation was only 2.3% without any time lag to reach this quality level. The proportion of invalid samples is usually much larger, even in the less challenging setting of studies in adult patients (Ng et al., 2017; Gioia et al., 2016). Whether this observation is an effect of the simulation intervention cannot be clarified due to the missing control group. However, the results are remarkable, given the fact that one third of pediatric trials are delayed due to poor recruitment, one fifth is discontinued (Pica and Bourgeois, 2016) and an optimum of protocol compliance and documentation correctness is normally reached after enrollment of 20–80% of participants (Taekman et al., 2010). We were very aware of this limitation when planning and conducting the intervention and its evaluation; however, we could not risk withholding the potential benefits of the intervention from parts of our sites.

## conclusion

Our innovative training concept to optimize the informed consent process and PK/PD sampling was associated with successful recruitment and a high-quality study conduct with a complex study protocol and in a very challenging population. The strengths of this approach include the recognition of weaknesses in processes and documents as well as the opportunity to identify and practice critical procedures before the study initiation. The effort to bring together a faculty of experts for simulation-based training, clinical trial experts and patient advocates seemed to pay off in our setting. It may be a very valuable complementary instrument to other methods for preparing study sites also in other challenging study settings. The developing process of this training intervention as described in this manuscript can be used as a template by other research consortia or study sponsors. The preventable mistakes identified in our trainings maybe relevant for other studies. Therefore, although a confirmation of the specific training effects in comparison to other or no intervention was not feasible in our setting, our results may ultimately help to improve the preparedness of study sites in other trials, particularly in the challenging setting of pediatric populations.

## Data Availability Statement

The raw data supporting the conclusions of this article will be made available by the authors, without undue reservation.

## Ethics Statement

The studies involving human participants were reviewed and approved by Ethics Committee of Salzburg state government (study no. 415-E/1909/2-2015) and the Ethics Committee of the Heinrich Heine University Duesseldorf (study no. 5138). The patients/participants provided their written informed consent to participate in this study.

## Author Contributions

BB, AC, AL, IK, SL, KK, and FL developed the idea and designed the training. LA, MD, MĐ, JB, MM, and VS contributed to the design. BB, AC, AL, and FL conducted the simulation training, performed the survey and analysed the data. HS assisted with the statistical analysis. BB, AC, AL, and FL drafted the manuscript. All authors provided critical feedback and helped shape the research, analysis and manuscript.

## Funding

The activities within the simulation training has received funding from the European Union Seventh Framework Programme (FP7/2007‐2013) under grant agreement n 602295 (LENA).

## Conflict of Interest

IK is the owner of Pharmaplex BVBA, an SME consortium partner of the LENA project. AL is the owner of Pharmabrain Research and Training Center.

The remaining authors declare that the research was conducted in the absence of any commercial or financial relationships that could be construed as a potential conflict of interest.

## References

[B1] Bajcetic M. Wildt S. N. de. Dalinghaus M. Breitkreutz J. Klingmann I. Lagler F. B. (2019). Orodispersible minitablets of enalapril for use in children with heart failure (LENA): rationale and protocol for a multicentre pharmacokinetic bridging study and follow-up safety study. Contemp. Clin. Trials Commun. 15, 100393 10.1016/j.conctc.2019.100393 31249901 PMC6586986

[B2] Caldwell P. H. Butow P. N. Craig J. C. (2003). Parents' attitudes to children's participation in randomized controlled trials. J. Pediatr. 142, 554–559. 10.1067/mpd.2003.192 12756389

[B3] Chopra V. Gesink B. J. de Jong J. Bovill J. G. Spierdijk J. Brand R. (1994). Does training on an anaesthesia simulator lead to improvement in performance?. Br. J. Anaesth. 73, 293–297. 10.1093/bja/73.3.293 7946851

[B4] Ciplea A. M. Laeer S. Burckhardt B. B. (2018). A feasibility study prior to an international multicentre paediatric study to assess pharmacokinetic/pharmacodynamic sampling and sample preparation procedures, logistics and bioanalysis. Contemp Clin Trials Commun 12, 32–39. 10.1016/j.conctc.2018.08.008 30225392 PMC6139604

[B5] Denhoff E. R. Milliren C. E. de Ferranti S. D. Steltz S. K. Osganian S. K. (2015). Factors associated with clinical research recruitment in a pediatric academic medical center–A web-based survey. PLoS One 10, e0140768 10.1371/journal.pone.0140768 26473602 PMC4608599

[B6] Eppich W. Cheng A. (2015). Promoting Excellence and Reflective Learning in Simulation (PEARLS): development and rationale for a blended approach to health care simulation debriefing. Simulat. Healthc. J. Soc. Med. Simulat. 10, 106–115. 10.1097/SIH.0000000000000072 25710312

[B7] Gioia L. C. Kate M. Sivakumar L. Hussain D. Kalashyan H. Buck B. (2016). Early rivaroxaban use after cardioembolic stroke may not result in hemorrhagic transformation: a prospective magnetic resonance imaging study. Stroke 47, 1917 10.1161/STROKEAHA.116.013491 27222524

[B8] Glaser J. Nouri S. Fernandez A. Sudore R. L. Schillinger D. Klein-Fedyshin M. (2020). Interventions to improve patient comprehension in informed consent for medical and surgical procedures: an updated systematic review. Med. Decis. Making 40, 119–143. 10.1177/0272989X19896348 31948345 PMC7079202

[B9] Kasenda B. Elm E. von. You J. Blümle A. Tomonaga Y. Saccilotto R. (2014). Prevalence, characteristics, and publication of discontinued randomized trials. J. Am. Med. Assoc. 311, 1045–1051. 10.1001/jama.2014.1361 24618966

[B10] Kinnersley P. Phillips K. Savage K. Kelly M. J. Farrell E. Morgan B. (2013). Interventions to promote informed consent for patients undergoing surgical and other invasive healthcare procedures. Cochrane Database Syst. Rev., CD009445 10.1002/14651858.CD009445.pub2 23832767 PMC11663509

[B11] Kolb D. A. (2014). Experiential learning: experience as the source of learning and development. Upper Saddle River, NJ: Pearson Education, 1.

[B12] Koyfman S. A. Reddy C. A. Hizlan S. Leek A. C. Kodish A. E. (2016). Informed consent conversations and documents: a quantitative comparison. Cancer 122, 464–469. 10.1002/cncr.29759 26505269 PMC4724216

[B13] Lagler F. B. Weineck S. B. Schwab M. (2015). Enduring and emerging challenges of informed consent. N. Engl. J. Med. 372, 2170–2171. 10.1056/NEJMc1503813 26017841

[B14] Liénard J.-L. Quinaux E. Fabre-Guillevin E. Piedbois P. Jouhaud A. Decoster G. (2006). Impact of on-site initiation visits on patient recruitment and data quality in a randomized trial of adjuvant chemotherapy for breast cancer. Clin. Trials 3, 486–492. 10.1177/1740774506070807 17060222

[B15] Lippi G. Simundic A.-M. Rodriguez-Manas L. Bossuyt P. Banfi G. (2016). Standardizing *in vitro* diagnostics tasks in clinical trials: a call for action. Ann. Transl. Med. 4, 181 10.21037/atm.2016.04.10 27275494 PMC4876285

[B16] Mason S. A. Allmark P. J. (2000). Obtaining informed consent to neonatal randomised controlled trials: interviews with parents and clinicians in the Euricon study. Lancet 356, 2045–2051. 10.1016/s0140-6736(00)03401-2 11145490

[B17] McGaghie W. C. Issenberg S. B. Cohen E. R. Barsuk J. H. Wayne D. B. (2011). Does simulation-based medical education with deliberate practice yield better results than traditional clinical education? A meta-analytic comparative review of the evidence. Acad. Med. 86, 706–711. 10.1097/ACM.0b013e318217e119 21512370 PMC3102783

[B18] Menon K. Ward R. E. Gaboury I. Thomas M. Joffe A. Burns K. (2012). Factors affecting consent in pediatric critical care research. Intensive Care Med. 38, 153–159. 10.1007/s00134-011-2412-0 22120768

[B19] Mitchell D. J. Edward Russo J. Pennington N. (1989). Back to the future: temporal perspective in the explanation of events. J. Behav. Decis. Making 2, 25–38. 10.1002/bdm.3960020103

[B20] Mitchell E. J. Godolphin P. J. Meakin G. Sprange K. (2020). Do investigator meetings improve recruitment rates in clinical trials? A retrospective before-and-after study of data from nine multi-centre clinical trials. Trials 21, 514 10.1186/s13063-020-04465-1 32522228 PMC7288550

[B21] Ng C. S. Zhang Z. Lee S. I. Marques H. S. Burgers K. Su F. (2017). CT perfusion as an early biomarker of treatment efficacy in advanced ovarian cancer: an ACRIN and GOG study. Clin. Cancer Res. 23, 3684–3691. 10.1158/1078-0432.CCR-16-1859 28174234 PMC5720368

[B22] Pica N. Bourgeois F. (2016). Discontinuation and nonpublication of randomized clinical trials conducted in children. Pediatrics, 138, 138 10.1542/peds.2016-0223 PMC5005019 27492817

[B23] Samiee-Zafarghandy S. Mazer-Amirshahi M. van den Anker J. N. (2014). Trends in paediatric clinical pharmacology data in US pharmaceutical labelling. Arch. Dis. Child. 99, 862–865. 10.1136/archdischild-2013-305605 25063835

[B24] Schandelmaier S. Tomonaga Y. Bassler D. Meerpohl J. J. Elm E. von. You J. J. (2017). Premature discontinuation of pediatric randomized controlled trials: a retrospective cohort study. J. Pediatr. 184, 209–214.e1. 10.1016/j.jpeds.2017.01.071 28410086

[B25] Schwid H. A. Rooke G. A. Carline J. Steadman R. H. Murray W. B. Olympio M. (2002). Evaluation of anesthesia residents using mannequin-based simulation: a multiinstitutional study. Anesthesiology 97, 1434–1444. 10.1097/00000542-200212000-00015 12459669

[B26] Shapiro M. J. (2004). Simulation based teamwork training for emergency department staff: does it improve clinical team performance when added to an existing didactic teamwork curriculum?. Qual. Saf. Health Care 13, 417–421. 10.1136/qshc.2003.005447 15576702 PMC1743923

[B27] Taekman J. M. Stafford-Smith M. Velazquez E. J. Wright M. C. Phillips-Bute B. G. Pfeffer M. A. (2010). Departures from the protocol during conduct of a clinical trial: a pattern from the data record consistent with a learning curve. Qual. Saf. Health Care 19, 405–410. 10.1136/qshc.2008.028605 20702441 PMC3258507

[B28] Treasure T. Morton D. (2012). GRIST: growing recruitment in interventional and surgical trials. J. R. Soc. Med. 105, 140–141. 10.1258/jrsm.2011.110307 22532650 PMC3343707

[B29] Treweek S. Pitkethly M. Cook J. Fraser C. Mitchell E. Sullivan F. (2018). Strategies to improve recruitment to randomised trials. Cochrane Database Syst. Rev. 2, MR000013 10.1002/14651858.MR000013.pub6 29468635 PMC7078793

[B30] Windsor J. A. Sturm L. P. Cosman P. H. Cregan P. Hewett P. J. Maddern G. J. (2008). A systematic review of skills transfer after surgical simulation training. Ann. Surg. 248, 166 10.1097/SLA.0b013e318188434710.1097/SLA.0b013e318176bf24 18650625

[B31] Wright M. C. Taekman J. M. Barber L. Hobbs G. Newman M. F. Stafford-Smith M. (2005). The use of high-fidelity human patient simulation as an evaluative tool in the development of clinical research protocols and procedures. Contemp. Clin. Trials 26, 646–659. 10.1016/j.cct.2005.09.004 16226924

