# Supplementary material for: Simulation Training to Improve Informed Consent and Pharmacokinetic/Pharmacodynamic Sampling in Pediatric Trials
Source: Front Pharmacol. 2020 Dec 11;11:603042. doi: 10.3389/fphar.2020.603042 (PMC7793942; doi:10.3389/fphar.2020.603042)
Supplement: Supplementary file 1 [file datasheet1.pdf]

Supplemental material 1: Adopted experiential learning cycle of the communication module (A) and PK/PD sampling (B) according to Kolb (17).

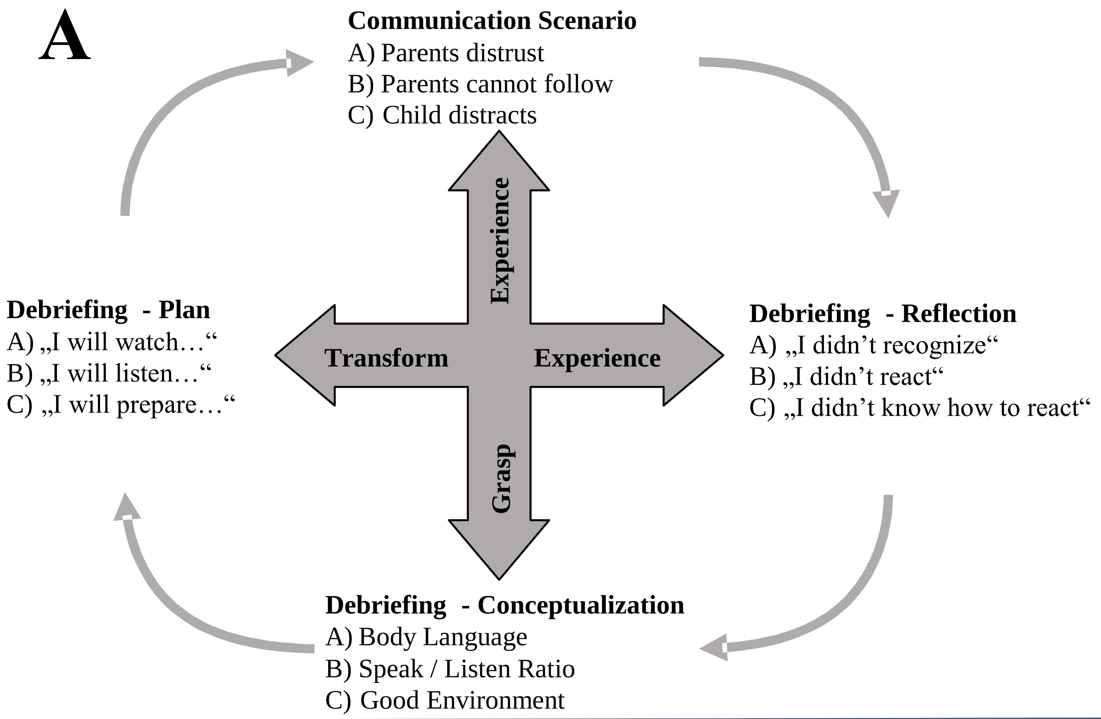

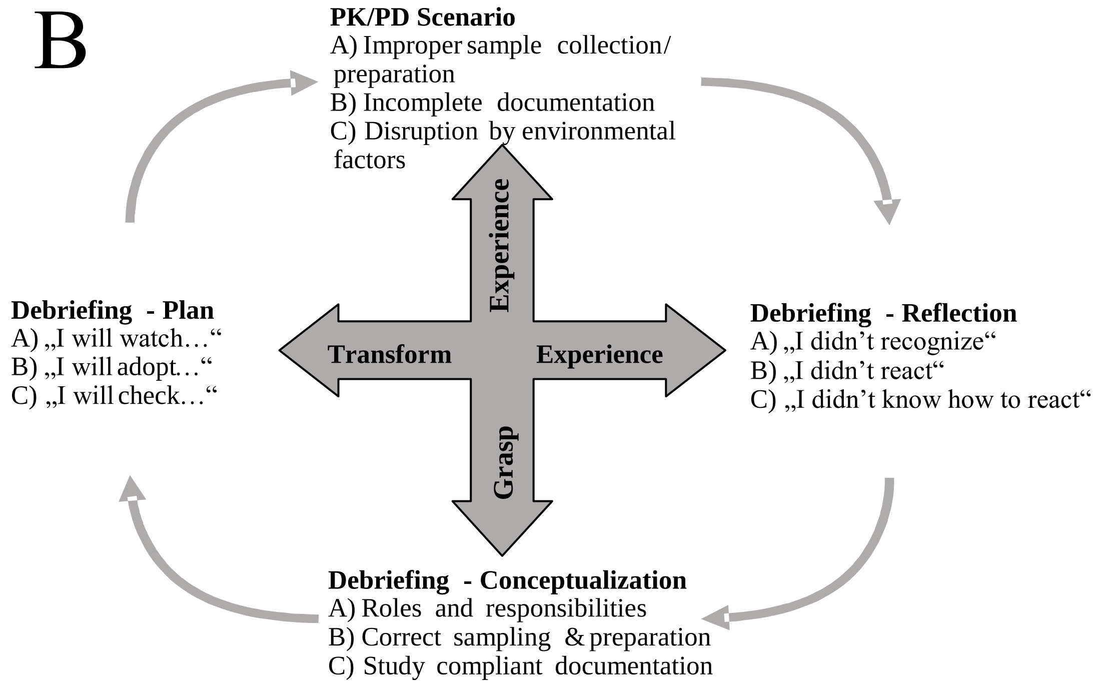

Supplemental material 2: Training concept of the LENA project.

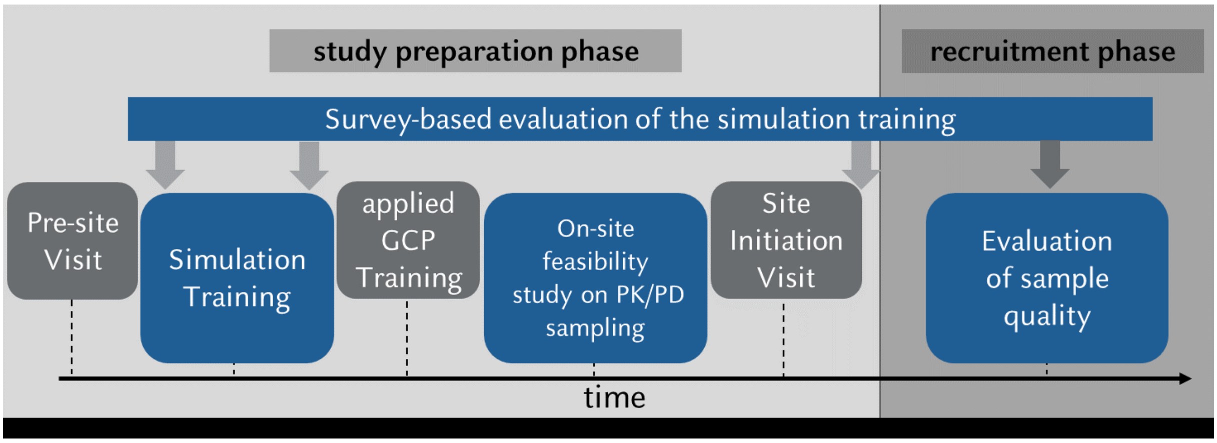

Concepts and training in blue mark additional training within the LENA wide training concept, while grey boxes indicate regular study-related visits/training and assessments.

Supplemental material 3: Questionnaires

Questionnaire 1 – distributed to participants after a general introductory presentation and before the simulation training, but before the introductory presentation on PK/PD sampling

Scale for questions 1-4:	5 point Likert scale (very poor/poor/sufficient/good/very good)
Scale for question 5:	5 point Likert scale (not relevant/ of little relevance/ neither relevant nor irrelevant/relevant/very relevant)

1. How familiar are you with the core elements of the upcoming clinical trials? (e.g. inclusion/exclusion criteria, study procedures)
2. How would you rate your ability to communicate these core elements appropriately to the different target audiences? (e.g. patients, parents and colleagues)
3. How well are you prepared to deal with challenging communicative situations, which typically occur during a clinical study with either patients, parents or colleagues?
4. How would you rate your ability to successfully perform the sampling and sample processing related procedures?
5. You have already been informed about the Simulation-trainings main aims and elements. How useful are the training topics on study preparation regarding the upcoming paediatric studies?

Questionnaire 2 – Distributed after the last round of training on PK/PD

Scale for questions 1-5:	5-point Likert scale (very poor/poor/sufficient/good/very good)
Scale for question 6:	5-point Likert scale (not relevant/ of little relevance/ neither relevant nor irrelevant/relevant/very relevant)
Scale for questions 7-12:	5-point Likert scale (strongly agree/disagree/undecided/agree/strongly agree)

1. How would rate your ability to communicate the core elements of the clinical trials appropriately to the different target audiences? (e.g. patients, parents and colleagues)?
2. How well are you prepared to deal with challenging communicative situations, which typically occur during a clinical study with either patients, parents or colleagues?
3. How would you rate your ability to successfully perform the sampling and sample processing related procedures?
4. How do you rate your own performance during the focused training on communication with regard to the following criteria?
   4.1 Completeness of relevant information communicated to a discussion partner
   4.2 Correctness of information transmitted to the discussion partner
   4.3 General comprehensibility
5. How do you rate your performance during the focused training on sampling and sample preparation concerning the following criteria?
   5.1 Completeness of procedures
   5.2 Correctness of performed procedures
   5.3 Speed/ duration of performed procedures
   5.4 Overall confidence during sample handling

6. How useful are the training topics on study preparation regarding the upcoming pediatric trials?
7. Did you feel stressed during the focused scenarios on Communication?
8. Did you feel stressed during the focused scenarios on sampling and sample preparation?
9. Did the training have a positive impact on your knowledge regarding the trained topics?
10. Did the training have a positive impact on your attitude regarding the trained topics?
11. Will the training have an impact on your mode of practice?
12. Do you think that simulation scenarios are a suitable approach in training complex study-related procedures and capabilities?

Questionnaire 3 – Distributed before study start

Scale for questions 1-7:	5-point Likert scale strongly agree/disagree/undecided/agree/strongly agree)
Scale for question 8:	5-point Likert scale (not relevant/ of little relevance/neither relevant nor irrelevant/relevant/very relevant)

1. Did the training have a positive effect on your preparation for the study?
2. Did the experiences from the simulation-training have an impact on your preparation procedures?
3. Did the training have a positive impact on your knowledge regarding the trained topics?
4. Did the training have a positive impact on your attitude regarding the trained topics?
5. Will the training have an impact on your mode of practice?
6. Do you think the simulation scenarios are a suitable approach in training complex study-related procedures and abilities?
7. Do you think the performance during the study will benefit from the simulation-training?
8. How useful are the training topics on study preparation regarding the upcoming pediatric studies?

Questionnaire 4 – Distributed during the recruitment

Scale for questions 1-6:	5-point Likert scale strongly agree/disagree/undecided/agree/strongly agree)
Scale for question 7:	5-point Likert scale (not relevant/of little relevance/neither relevant nor irrelevant/relevant/very relevant)

1. Do you think your performance during the study benefited from the training?
2. Did the training have a positive impact on your knowledge regarding the trained topics?
3. Did the training have a positive impact on your attitude regarding the trained topics?
4. Will the training have an impact on your mode of practice?
5. Do you think the simulation scenarios are a suitable approach in training complex study-related procedures and capabilities?

6. Would you recommend simulation-based training on the most challenging aspects of a clinical study as part of the preparation procedures?
7. How useful are the training topics on study preparation regarding the upcoming pediatric studies?

Supplemental material 4: Demographics of participants

Gender	10 male	43.5%
	13 female	56.5%
Nationality	6 Dutch	26.0%
	3 British	13.0%
	4 Hungarian	17.5%
	3 Austrian	13.0%
	7 Serbian	30.5%
Profession	13 medical doctors	56.5%
	10 study nurses	43.5%
Years of experience	<5 years	39.1%
	5 years and more	60.9%
Total	23 participants	
